# A Three-Dimensional Target Depth-Resolution Method with a Single-Vector Sensor

**DOI:** 10.3390/s18041182

**Published:** 2018-04-12

**Authors:** Anbang Zhao, Xuejie Bi, Juan Hui, Caigao Zeng, Lin Ma

**Affiliations:** 1 Acoustic Science and Technology Laboratory, Harbin Engineering University, Harbin 150001, China; zhaoanbang@hrbeu.edu.cn (A.Z.); bixuejie@hrbeu.edu.cn (X.B.); cgzeng@hrbeu.edu.cn (C.Z.); malin@hrbeu.edu.cn (L.M.); 2 Key Laboratory of Marine Information Acquisition and Security, Harbin Engineering University, Ministry of Industry and Information Technology, Harbin 150001, China; 3 College of Underwater Acoustic Engineering, Harbin Engineering University, Harbin 150001, China; 4 National Key Laboratory of Science and Technology on Underwater Acoustic Antagonizing, China State Shipbuilding Corporation Systems Engineering Research Institute, Beijing 100036, China

**Keywords:** aerial target, depth resolution, three-dimensional, multi-target, Monte Carlo

## Abstract

This paper mainly studies and verifies the target number category-resolution method in multi-target cases and the target depth-resolution method of aerial targets. Firstly, target depth resolution is performed by using the sign distribution of the reactive component of the vertical complex acoustic intensity; the target category and the number resolution in multi-target cases is realized with a combination of the bearing-time recording information; and the corresponding simulation verification is carried out. The algorithm proposed in this paper can distinguish between the single-target multi-line spectrum case and the multi-target multi-line spectrum case. This paper presents an improved azimuth-estimation method for multi-target cases, which makes the estimation results more accurate. Using the Monte Carlo simulation, the feasibility of the proposed target number and category-resolution algorithm in multi-target cases is verified. In addition, by studying the field characteristics of the aerial and surface targets, the simulation results verify that there is only amplitude difference between the aerial target field and the surface target field under the same environmental parameters, and an aerial target can be treated as a special case of a surface target; the aerial target category resolution can then be realized based on the sign distribution of the reactive component of the vertical acoustic intensity so as to realize three-dimensional target depth resolution. By processing data from a sea experiment, the feasibility of the proposed aerial target three-dimensional depth-resolution algorithm is verified.

## 1. Introduction

Current acoustic positioning technology operates mainly through the processing of signals collected by underwater sensors. Based on the processing results, the three-dimensional positioning of the targets can be realized through the estimation of distance, depth and azimuth of the targets.

The resolution of surface and underwater targets is one of the hotspots in the field of underwater acoustic detection. It can effectively improve the resolution accuracy of underwater threat-representative targets so as to improve the safety and concealment of underwater maneuvering targets. The target depth estimation is the key problem of target category resolution, while the effective resolution of surface and underwater targets is the key problem of the target depth estimation. The effective resolution of surface and underwater targets has not yet been successfully applied. A great deal of relevant research work is underway at home and abroad in this area.

A target detection and localization algorithm is proposed by Bucker [1] in which the measured cross-spectrum matrix is compared to a matrix calculated for a target at an assumed location. Simulation examples are shown in a shallow-water environment. Hinich [2] proposed a method in which the maximum likelihood estimator is used to estimate the target depth based on the vertical array in an infinite horizontal waveguide (such as the ocean). The accuracy of the above method is limited by the number of modes detected by the array, regardless of the array size. Shang [3] proposed an approach for target depth estimation in a waveguide based on the mode filtering technique. Some numerical examples and scale-model experimental results are given. Yang [4] adopted a method based on eigenvector decomposition technique to extract the mode amplitudes for data received by a vertical array. The correlation of the measured (decomposed) mode amplitudes with the theoretically calculated mode amplitudes is maximum at the target depth. The data-based method for moving target depth estimation is also demonstrated experimentally [5]. Goldhahn [6] proposed a waveguide invariant depth classification method based on adaptive matched-filtering under uncertain environmental conditions. Matched field processing (MFP) [7,8,9,10,11,12,13,14] is a generalized beamforming method which uses the spatial complexities of acoustic fields in an ocean waveguide to estimate the range, depth and azimuth of targets.

Many native and foreign scholars have done much research work on the resolution of surface and underwater targets.

Premus [15] investigated an application of the Scharf–Friedlander matched subspace detector [16] to the problem of trapped mode subspace discrimination in a shallow-water waveguide. He also proposed a method in which a mode-filter beamformer is combined with different decision metrics for the purpose of the target depth discrimination. The concept is based on the premise that deep and shallow targets robustly separate in mode space [17].

With the progress of shock absorption and noise reduction technology, the radiated noise of underwater moving platform in the high frequency band is lower and lower. Scholars such as Kuperman and D’Spain [18,19,20,21,22,23] have carried out a lot of research work in the field of ocean acoustic interference phenomena and signal processing. Brekhovskikh and Lysanov et al. [24,25,26] defined an important scalar parameter of acoustic waveguide interference structure (waveguide invariant) and described the nature and application of the waveguide invariant.

In order to solve the problem of target depth resolution in the very low frequency field, many scholars like Hui and Yu et al. [27,28,29,30,31] proposed a cross-spectrum (between pressure and velocity) signal-processing algorithm to distinguish the surface targets and the underwater targets. Both the active component of the horizontal interactive complex acoustic intensity and the reactive component of the vertical interactive complex acoustic intensity in the low-frequency field can be used to identify target depth.

Researchers such as An [32], Premus [33] and Creamer [34] introduced a way to define the modified modal scintillation index (MMSI). It has been proved in an analytical form that the index MMSI is depth-dependent and independent of source level and source range under the condition of the ideal waveguide. The probability density functions (PDFs) of different normal modes’ MMSI of surface and underwater targets are different so that the PDFs can be used to separate the surface and underwater targets. Mitchell [35] studied a method to determine the spectrum characteristic period by using power cepstrum techniques so as to estimate the actual target depth.

Above all, for the depth resolution of surface and underwater targets, domestic and foreign scholars conducted some research and achieved some results. Nevertheless, previous research work mainly focused on the target category resolution in a single-target case mostly from a theoretical dimension and lacked data verification. Moreover, there are still few studies on the target category-resolution methods in multi-target cases. In addition, the researches on target depth resolution are mainly a binary decision; there is little research on the target depth resolution of the aerial targets.

The novelty of this paper lies mainly in the following several aspects:

1. Based on the obtained target depth-resolution and bearing-time recording information, the target category and number resolution in single-target and multi-target cases are realized. Hence, the distinction between the single-target multi-line spectrum case and the multi-target multi-line spectrum case can be realized. On the foundation of the theoretical research, the proposed algorithm is verified by Monte Carlo simulation for the first time. The proposed target number and category-resolution method in the case of a multi-target can be applied in underwater platform signal-processing devices to enhance its safety and concealment.
2. In this paper, the depth-resolution method of the aerial targets is firstly proposed, which is verified by theoretical derivation and processing of sea experiment data. Hence, the three-dimensional target depth resolution is achieved.
3. The previous azimuth-estimation algorithm is mainly aimed at the azimuth-estimation requirement in a single-target case. The improved azimuth-estimation algorithm proposed in this paper is more suitable for multi-target cases.

## 2. Theory and Model

### 2.1. Normal-Mode Expressions of the Field in Shallow Water

#### 2.1.1. Field Excited by Surface or Underwater Targets

Bucker has given the normal-mode expressions for a source and receiver in an arbitrarily stratified ocean [36]. Bucker considered an ocean with a depth-dependent sound-velocity profile $c_{w}(z)$ and a harmonic source of angular frequency ω, so the acoustic wavenumber in the ocean is $k_{w}(z)=\omega /c_{w}(z)$. The acoustic pressure *P*(*r*,*z*) at depth *z* due to a unit source at range *r* and depth $z_{0}$ is the solution to the Helmholtz equation. The equation is as follows:

(1) $$\nabla^{2}P+k_{w}^{2}P=-4\pi \delta (R)$$

in which δ is the delta function, and $R=\sqrt{r^{2}+{\left(z-z_{0}\right)}^{2}}$. If the water were homogeneous and infinite, the solution should be $P=e^{jk_{w}R}/R$, representing spherical waves. Note that we have dropped explicit references to the time factor $e^{-j\omega t}$.

We rearrange Bucker’s notation slightly to produce the normal-mode sum [37]. The normal mode expression of the pressure field excited by surface or underwater targets is:

(2) $$p(r,z)=j\pi \sum_{n}\Psi_{n}(z_{0})\Psi_{n}(z)H_{0}^{\left(1\right)}(\xi_{n}r)$$

in which, n is the serial number of the normal mode, $\Psi_{n}(z_{0})$ is the eigenfunction at the source position, $\Psi_{n}(z)$ is the eigenfunction at the receiver position, $\Psi_{n}(z)=\sin(\beta_{1n}z)$, $\beta_{in}=\sqrt{k_{i}^{2}-\xi_{n}^{2}}$, $k_{i}=\omega /c_{i}$(i=0,1,2), $\xi_{n}$ is the *n*-th order eigenvalue, and $H_{0}^{\left(1\right)}$ is the Hankel function of first kind.

(3) $$p(r,z)=2\pi \omega \rho_{1}\sum_{n}A_{n}\sin(\beta_{1n}z)H_{0}^{1}(\xi_{n}r)=e^{-j\frac{\pi }{4}}\sqrt{\frac{8\pi }{r}}\omega \rho_{1}\sum_{n}\sqrt{\frac{1}{\xi_{n}}}A_{n}\sin(\beta_{1n}z)e^{j\xi_{n}r}$$

in which, $A_{n}=\frac{\Psi_{n}(z_{0})}{H-\frac{\Psi_{n}(2H)}{2\beta_{1n}}+b\frac{\Psi_{n}^{2}(H)}{j\beta_{2n}}}$, $b=\rho_{1}/\rho_{2}$. *H* is the sea depth. $c_{0}$ and $\rho_{0}$ are the sound velocity and density of the air layer respectively. $c_{1}$ and $\rho_{1}$ are the sound velocity and density of the water layer respectively. $c_{2}$ and $\rho_{2}$ are the sound velocity and density of the seabed medium layer respectively. The sea surface is an absolutely soft interface above which the pressure is zero.

The normal mode expression of the vertical velocity field excited by surface or underwater targets can be expressed as follows:

(4) $$v_{z}(r,z)=\pi \sum_{n}\Psi_{n}(z_{0})\Psi_{n}^{\prime }(z)H_{0}^{\left(1\right)}(\xi_{n}r)$$

(5) $$v_{z}(r,z_{0},z)=-je^{-j\frac{\pi }{4}}\sqrt{\frac{8\pi }{r}}\sum_{n}\sqrt{\frac{1}{\xi_{n}}}A_{n}\beta_{1n}\cos(\beta_{1n}z)e^{j\xi_{n}r}$$

#### 2.1.2. Field Excited by the Aerial Targets

If the aerial target is at height *h* in the homogeneous air layer, the normal mode expression of the pressure field excited by the aerial targets can be represented as:

(6) $$p(r,z)=-\pi \sum_{n}\frac{1}{\beta_{0n}}e^{j\beta_{0n}h}\Psi_{n}^{\prime }(0)\Psi_{n}(z)H_{0}^{\left(1\right)}(\xi_{n}r)$$

in which $\Psi_{n}^{\prime }(0)$ is the derivative of the *n*-th normal mode function at the sea surface. The excitation coefficient of the *n*-th normal mode is:

(7) $$\Psi_{n}(h)=j\frac{1}{\beta_{0n}}e^{j\beta_{0n}h}\Psi_{n}^{\prime }(0)$$

The expression of the pressure field excited by the aerial targets can be changed to:

(8) $$p(r,z)=j\pi \sum_{n}\Psi_{n}(h)\Psi_{n}(z)H_{0}^{\left(1\right)}(\xi_{n}r)$$

Comparing (2) with (8), it can be found that the only difference between the normal mode excited by the aerial targets and the normal mode excited by the surface targets is the different excitation coefficient. The target height only affects the phase of the excitation coefficient and does not affect the amplitude of the excitation coefficient.

When the surface target’s depth satisfies $z_{0}<<\lambda$ (λ is the wavelength in the water), $\Psi_{n}(z_{0})\approx \Psi_{n}(0)+\Psi_{n}^{\prime }(0)z_{0}$, then [38]: $\Psi_{n}^{\prime }(0)\approx \frac{\Psi_{n}(z_{0})}{z_{0}}$. Since $c_{0}<<c(z)$ (c(z) is the sound velocity in the water), the eigenvalue satisfies $\xi_{n}<<k_{0}$. The vertical wavenumber $\beta_{0n}$ in the air can be approximate to $\beta_{0n}=\sqrt{k_{0}^{2}-\xi_{n}^{2}}\approx k_{0}-\frac{\xi_{n}^{2}}{2k_{0}}\approx k_{0}$. When the aerial target’s height satisfies $h<\frac{\frac{1}{4}\lambda_{0}}{{\left(\frac{c_{0}}{c_{1}}\right)}^{2}-{\left(\frac{c_{0}}{c_{\max}}\right)}^{2}},c_{\max}=\max(c_{1},c_{2})$, $\lambda_{0}$ is the wavelength in the air, then $e^{j\beta_{0n}h}\approx e^{ik_{0}h}e^{-i\xi_{n}^{2}h/2k_{0}}\approx 1$. Thus, $\Psi_{n}(h)=j\frac{\Psi_{n}(z_{0})}{k_{0}z_{0}}$. Then the expression of p(r,z) can be changed to:

(9) $$p(r,z)=-\frac{\pi }{k_{0}z_{0}}\sum_{n}\Psi_{n}(z_{0})\Psi_{n}(z)H_{0}^{\left(1\right)}(\xi_{n}r),(h\leq 0,0\leq z\leq H)$$

Through a comparison of (2) and (9), it can be found that the amplitude difference between the pressure field excited by the aerial targets and that excited by the surface targets is a multiple of $k_{0}z_{0}$ in the shallow water.

In the same way, the expression of the vertical velocity field excited by the aerial targets is:

(10) $$v_{z}(r,z_{0},z)=j\frac{\pi }{k_{0}z_{0}}\sum_{n}\Psi_{n}(z_{0})\Psi_{n}^{\prime }(z)H_{0}^{\left(1\right)}(\xi_{n}r),(h\leq 0,0\leq z\leq H)$$

### 2.2. Three-Dimensional Target Depth-Resolution Method

#### 2.2.1. Target Depth-Resolution Method for Surface and Underwater Targets

The vertical complex acoustic intensity of the normal mode is expressed as [39]:

(11) $$I_{z}(r,\omega )=p(r,\omega )\cdot v_{z}^{*}(r,\omega )$$

In the formula, ω represents the frequency, the superscript * represents the complex conjugate operation, p(r,ω) and $v_{z}(r,\omega )$ are the Fourier transform of p(r,t) and $v_{z}(r,t)$.

The complex acoustic intensity consists of the sum of two components, active and reactive acoustic intensity, as follows:

(12) $$I_{z}(r,\omega )=I_{zA}(r,\omega )+jI_{zR}(r,\omega )$$

In the formula, $I_{zA}(r,\omega )$ is called active acoustic intensity, which means the energy flux that can propagate to the distance; $I_{zR}(r,\omega )$ is called reactive acoustic intensity, which means the energy flux that does not propagate.

Substituting (3) and (5) to (11), the expression of the vertical complex acoustic intensity is:

(13) $$\begin{array}{l} I_{z}=pv_{z}^{*}\;\approx j\frac{8\pi \omega \rho_{1}}{r}\sum_{n}\frac{\beta_{1n}}{\xi_{n}}\sin(\beta_{1n}z)\cos(\beta_{1n}z)A_{n}^{2}\\ \;+j\frac{8\pi \omega \rho_{1}}{r}\sum_{n,n\neq m}\sum_{m}\frac{\beta_{1m}}{\sqrt{\xi_{n}\xi_{m}}}\;\sin(\beta_{1n}z)\cos(\beta_{1m}z)A_{n}A_{m}\{\cos[(\xi_{m}-\xi_{n})r]+j\sin[(\xi_{m}-\xi_{n})r]\} \end{array}$$

The active ($I_{zA}$) and reactive ($I_{zR}$) components of vertical complex acoustic intensity are:

(14) IzA=Re(pvz*)=8πωρ1r∑n,n≠m∑mβ1mξnξmsin(β1nz)cos(β1mz)AnAmsin[(ξm−ξn)r]

(15) IzR=Im(pvz*)=4πωρ1r{∑nβ1nξnsin(2β1nz)An2         +2 ∑n,n≠m∑mβ1mξnξmsin(β1nz)cos(β1mz)AnAmcos[(ξm−ξn)r]}

The target depth resolution is carried out by using the vertical complex acoustic intensity reactive component sign distribution to identify the target category (surface or underwater targets).

#### 2.2.2. Target Depth-Resolution Method for Aerial Targets

Assuming that $p_{0}(r,\omega )$ and $v_{z0}(r,\omega )$ are the frequency domain representations of the pressure and vertical velocity field excited by the aerial targets, respectively, p(r,ω) and $v_{z}(r,\omega )$ are the frequency domain representations of the pressure and vertical velocity field excited by the surface targets, respectively. According to (2), (4), (9) and (10):

(16) $$p_{0}(r,\omega )=\frac{j}{k_{0}z_{0}}p(r,\omega )$$

(17) $$v_{z0}(r,\omega )=\frac{j}{k_{0}z_{0}}v_{z}(r,\omega )$$

Substituting (16) and (17) to (11), we can get that the vertical complex acoustic intensity of aerial target is:

(18) $$I_{z0}(r,\omega )=p_{0}(r,\omega )\cdot v_{z0}^{*}(r,\omega )=\;(\frac{1}{k_{0}z_{0}}{)}^{2}p(r,\omega )\cdot v_{z}^{*}(r,\omega )$$

Through the contrast of (11) and (18), it can be found that there is only amplitude coefficient difference between the vertical complex acoustic intensity of aerial targets and that of surface targets. The coefficient difference ${\left(\frac{1}{k_{0}z_{0}}\right)}^{2}$ is a real number so as not to affect the sign distribution of the vertical complex acoustic intensity active and reactive components. Therefore, in the target depth-resolution process, the aerial targets can be regarded as a special case of the surface targets. Then, through the specific height estimation of the aerial targets [40], we can distinguish the aerial targets and the surface targets, if the receiver depth is known. The basic idea of the aerial target height-estimation method described in [40] is to calculate the distance between the source and receiver. Through the comparison of the calculated distance against the known receiver depth, the height estimation is realized. Combined with the calculated distance, whether the targets are aerial or surface targets depends on their calculated distances being smaller or larger than receiver depth.

### 2.3. Target Number-Resolution Method in Single-Target and Multi-Target Cases

This paper mainly aims to distinguish the single-target case and the multi-target case, so as to realize the target number resolution. The single-target multi-line spectrum and multi-target multi-line spectrum cases cannot be differentiated by time–frequency distribution, because there are many line spectrums on the time–frequency distribution in both cases, so that we cannot tell whether the case is single-target or multi-target. In this paper, we mainly make use of the azimuth information contained in the received signal to distinguish the single-target and multi-target cases. There is only one curve for the azimuth variant in the bearing-time recording of the single-target multi-line spectrum case, but there are multiple curves for the azimuth variant in the bearing-time recording of the multi-target multi-line spectrum case. Ideally, the number of curves for the azimuth variant is consistent with the target number. The so-called ideal case is that each curve for azimuth variant can be clearly identified.

#### 2.3.1. Azimuth-Estimation Method

**Figure 1:**
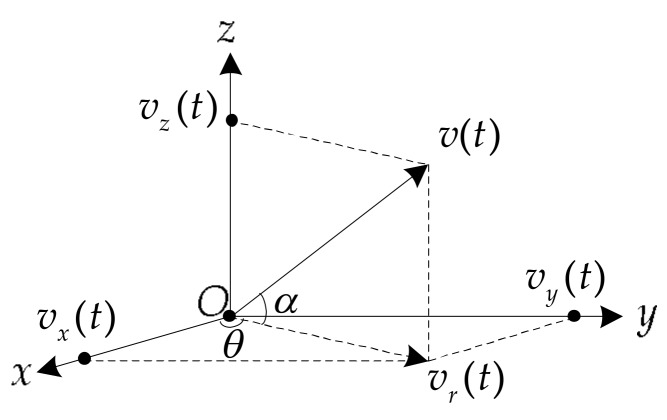
The geometric relationship between the velocity *v* and its three orthogonal components $n_{vx}(t)$, considering θ,α.

The vector sensor is the sensor used in the underwater vector field measurement, which is the combination of pressure sensor and velocity sensor (pressure gradient sensor, accelerometer, displacement meter, etc.) [41]. The vector sensor outputs the pressure signal p(t) and velocity signal v(t) which contains three orthogonal components $v_{x}(t),v_{y}(t),v_{z}(t)$. p(t)=x(t) and $\left[v_{x}(t),v_{y}(t),v_{z}(t)]=[v_{r}(t)\cos\theta ,v_{r}(t)\sin\theta ,v_{z}(t)]=\frac{1}{\rho c}x(t)[\cos\theta \cos\alpha ,\sin\theta \cos\alpha ,\sin\alpha \right]$, in which x(t) is the target signal. The geometric relationship between the orthogonal components is shown in Figure 1. θ is the horizontal azimuth angle (range: $0^{\circ }∼360^{\circ }$), the x-axis positive direction is $0^{\circ }$, α is the elevation angle (range: $-90^{\circ }∼90^{\circ }$), and the horizontal plane (*xoy* plane) is $0^{\circ }$ [42].

The expression of the aerial target-radiated noise refraction signal received by the sensor is: $\left[S_{p}(t),S_{vx}(t),S_{vy}(t),S_{vz}(t)]{=R}_{t}\cdot x(t)[1,\cos\theta \cos\alpha ,\sin\theta \cos\alpha ,\sin\alpha ]+[N_{p}(t),N_{vx}(t),N_{vy}(t),N_{vz}(t)\right]$. $S_{p}(t)$, $S_{vx}(t)$, $S_{vy}(t)$, $S_{vz}(t)$ are the pressure and velocity signals received by the vector sensor, $N_{p}(t),N_{vx}(t),N_{vy}(t),N_{vz}(t)$ are the isotropic noise components of pressure and velocity received by the vector sensor, and they are all independent of x(t). $R_{t}$ is the refraction coefficient, which meets: $R_{t}=\frac{2\rho_{1}c_{1}\sin\varphi_{0}}{\rho_{1}c_{1}\sin\varphi_{0}+\rho_{0}c_{0}\sin\varphi_{1}}$. $\varphi_{0}$ and $\varphi_{1}$ are the angles between the incident, refraction ray of the direct refraction wave and the target motion direction respectively.

The physical basis of complex acoustic intensity’s anti-interference performance is the correlation between pressure and velocity, whereas the pressure and velocity of isotropic environment interference are irrelevant or weakly correlated. The average output of complex acoustic intensity is:

(19) $$\left[\langle I_{x}(f)\rangle ,\langle I_{y}(f)\rangle ]=R_{t}^{2}[\langle S_{p}(f)S_{vx}^{*}(f)\rangle ,\langle S_{p}(f)S_{vy}^{*}(f)\rangle ]=R_{t}^{2}\langle {\left|X(f)\right|}^{2}\rangle [\cos\theta \cos\alpha ,\sin\theta \cos\alpha ]+\delta (f\right)$$

〈⋅〉 represents sliding-average periodgram operation. “*” represents complex conjugate, |⋅| represents the complex modulus calculation operation, and $S_{p}(f),S_{vx}(f),S_{vy}(f)$ are the Fourier transform of the pressure and velocity respectively, X(f) is the Fourier transform of the signal x(t), and δ(f) is the Fourier transform of the interference with the small quantity.

In the underwater acoustic channel, the acoustic Ohm law is approximately satisfied. Therefore, the pressure signal has the same phase as the velocity signal. According to the basic properties of the Fourier transform, the energy of the two signals in the same phase is concentrated on the real component of the cross spectrum. The imaginary component of the cross spectrum only contains the energy of interference and noise [42,43].

The cross spectrum method at a single frequency point is given by:

(20) θ(f)=tan−1{Re[〈Iy(f)〉]Re[〈Ix(f)〉]}

Re[⋅] represents the operation of obtaining the real component.

In this paper, we use the weighted bar graph method to do the statistical analysis of the azimuth-estimation results. The specific algorithm of the weighted bar graph method is given by:

(21) $$\zeta_{k}=\{\mathrm{mod}[\theta (f_{k}),2\pi ]\cdot 180/\pi \},(1\leq k\leq M)$$

(22) $$A(f_{k})=\sqrt{I_{x}^{2}(f_{k})+I_{y}^{2}(f_{k})}$$

(23) $$Ζ(n)=\sum_{k}A(f_{k})$$

ζ is the azimuth sequence in the angle domain; {⋅} represents the getting integer operation towards positive infinity; mod[⋅] represents the modulus operation; $\theta (f_{k})$ is the azimuth-estimation value at every frequency point; *k* is the sequence number of the frequency point; *M* is the total number of frequency points used in the azimuth estimation; $A(f_{k})$ is the complex acoustic intensity of the *k*-th frequency point; and *Z* is an array whose dimension is 1 × 360. We use the array *Z* to do the weighted statistic of the azimuth sequence ζ. The complex acoustic intensity $A(f_{k})$, used for summation in (23), meets $\zeta_{k}=n\;(n=1,2,\cdots ,360)$. *n* is an angle sequence that varies from 1° to 360°.

Using the azimuth-estimation method based on (20), we can calculate the azimuth value at every frequency point. Then, we use the weighted bar graph method to do a statistical analysis of the azimuth-estimation result at every frequency point, so as to get the probability distribution of the azimuth estimation at a certain time. The azimuth corresponding to the maximum value of the probability distribution is the desired target azimuth [40].

#### 2.3.2. Improved Azimuth-Estimation Method

The azimuth-estimation method described in Section 2.3.1 is effective in single-target cases and the azimuth-estimation accuracy is limited in multi-target cases, because signals radiated by multiple targets are superimposed together. In the azimuth-estimation process, a certain estimated target signal is disturbed by all the other target radiated signals, so that the azimuth-estimation effect in the whole frequency band is not very satisfactory. Aiming at the requirement of azimuth estimation in multi-target cases, the azimuth-estimation algorithm described in Section 2.3.1 is improved in this section. The improved azimuth-estimation method can still obtain the ideal azimuth-estimation results in multi-target cases. The difference between the improved and original azimuth-estimation method lies in the fact that in the multi-target cases, the azimuth estimation is not performed in the whole frequency band, but carried out at the frequency where the target signal has the highest line spectrum intensity after the frequency estimation based on the target time–frequency distribution.

In addition, in the case of multi-target, some target signals are stronger and some target signals are weaker. Assuming that the first target signal is strongest, the azimuth-estimation effect of the remaining targets may still be poor based on the improved azimuth-estimation method, because the remaining target signals are weaker signals relative to the first target signal. Ideally, the signal of the first target should not affect the azimuth estimation of other target-radiated signals. The azimuth-estimation results of other targets should not be within the range of the first target azimuth-estimation value $\pm 10^{\circ }$. However, in practice, the strong signal will have a great influence on the azimuth estimation of the weak signals, resulting in the azimuth-estimation results of the strong signals being contained in the azimuth-estimation results of the weak signals. To eliminate the influence of strong signals on weak signals, we add the threshold control. The threshold (that is, the permissible error of the azimuth estimation) is $\pm 10^{\circ }$. The specific steps of the added algorithm about the threshold control are as follows:

Step 1: Arrange the multiple target signals in descending order according to the line spectrum intensity of each signal;
Step 2: First, estimate the azimuth of the first target (namely, the target signal whose line spectrum intensity is strongest), and then obtain the final azimuth-estimation value after the statistical analysis, and the values in the range of the final azimuth-estimation value $\pm 10^{\circ }$ are considered as the azimuth-estimation results of the first target;
Step 3: When estimating the azimuth of the *i*-th target, the estimation results within the range at (*i*−1)-th target azimuth-estimation value $\pm 10^{\circ }$ should be cleared before the statistical analysis. *i* represents the serial number of the target, $i=2,\cdots ,M_{1}$, $M_{1}$ is the number of targets.
Step 4: Keep looping Step 3 until you have completed the azimuth estimation of all targets.

#### 2.3.3. Target Number-Resolution Method

**Figure 2:**
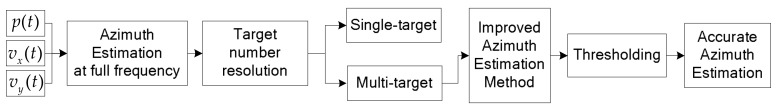
The flow chart of the target number-resolution process.

The target number-resolution process is shown in Figure 2. The azimuth-estimation method shown in Figure 2 is described in Section 2.3.1. The improved azimuth-estimation method is described in Section 2.3.2.

After using the received signal to carry out the azimuth estimation in the whole frequency band, if there is only one azimuth curve, we can judge that it is the single-target case. If there are many different azimuth curves, we can judge that it is the multi-target case, and then we use the improved azimuth-estimation method to obtain more precise azimuth-estimation results in the multi-target case.

## 3. Simulation Data and Results

All the marine environmental parameters of the simulations in this section are the same as shown in Table 1. However, the target simulation parameters in each subsection of this section are different, as shown later. The pressure and vertical velocity signals are all collected by a single three-dimensional vector sensor in the following simulations. In the following grayscale images, the lighter the color, the higher the data value.

### 3.1. Field Excited by Aerial and Surface Targets

Target is a point source which radiates a single-frequency harmonic sound wave whose frequency is *f* = 40 Hz. The other target simulation parameters are shown in Table 2.

**Figure 3:**
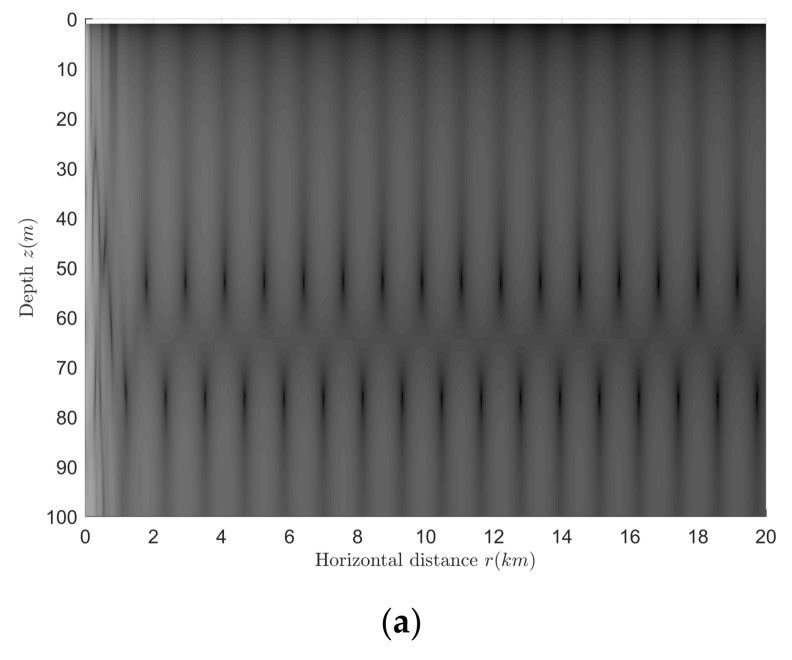

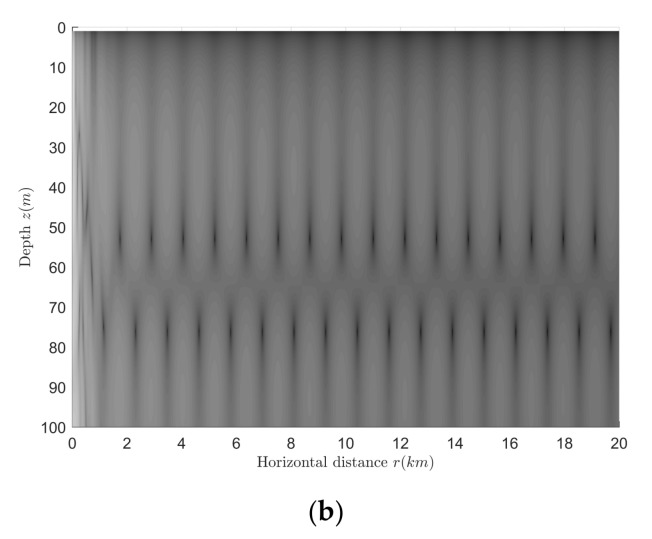
Comparison of the shallow-water field excited by an aerial target and its equivalent surface target. (**a**) The aerial target; (**b**) the equivalent surface target.

The shallow water pressure field excited by an aerial target and a surface target are shown in Figure 3a,b. The simulation results show that the aerial target field is similar to the equivalent surface target field, and the only difference between the above two fields is the amplitude. The mean value of the amplitude difference is 11.30 dB, while the theoretical difference value is $20\mathrm{lg}(k_{0}z_{0})=20\mathrm{lg}(\frac{2\pi f_{0}}{c_{0}}z_{0})\approx 11.51\;\mathrm{dB}$, both values are in good agreement, so the aerial target can be equivalent to a surface target.

### 3.2. Basic Theory Verification

#### 3.2.1. Harmonic Point Source

Target is a point source that radiates a single-frequency harmonic wave. The detailed target simulation parameters are shown in Table 3.

**Figure 4:**
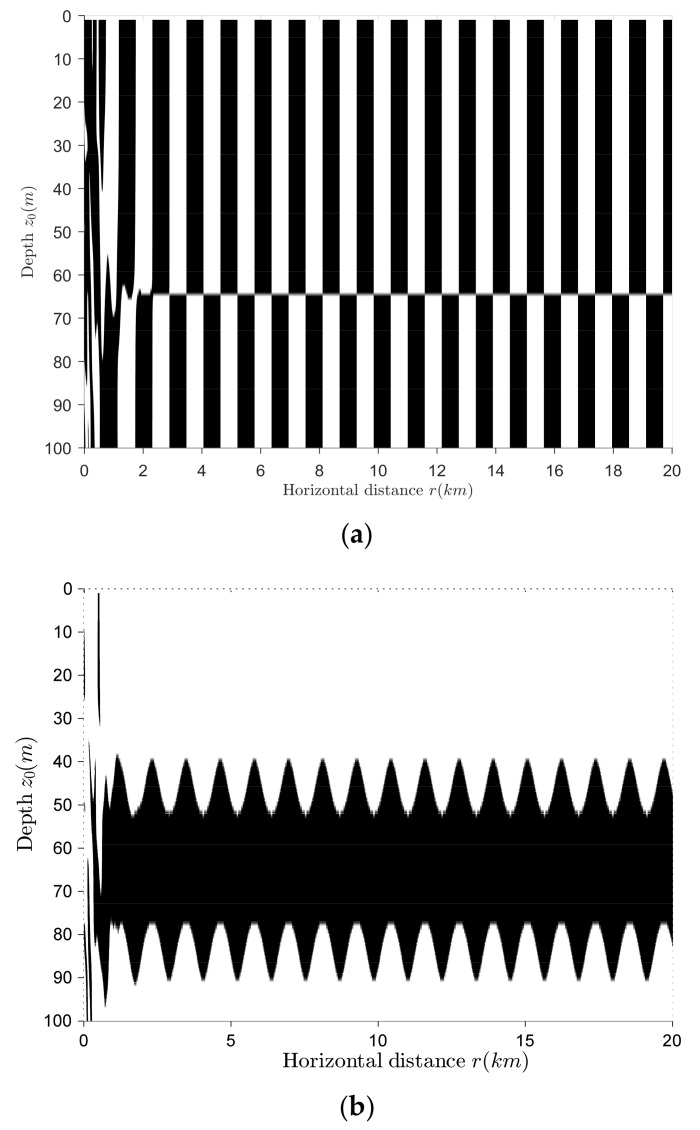
Sign distribution of the vertical complex acoustic intensity. (**a**) Active component; (**b**) reactive component.

The sign distributions of the vertical complex acoustic intensity active and reactive components are shown in Figure 4a,b respectively. Black means that the sign is negative and white means that the sign is positive. As can be seen from Figure 4a,b, the target depth can be identified by using the sign distribution of the vertical complex acoustic intensity reactive component. Under the assumption that no targets existed near the seabed, when the sign is positive, the target at this depth can be judged as a surface target; when the sign is negative, the target at this depth can be identified as an underwater target. The critical depth of surface and underwater targets is between 40 m and 53 m. The surface targets (whose depths are less than 40 m) and the underwater targets (whose depths are more than 53 m) have a better target category-resolution effect. The target category-resolution effect of the targets located in the depth range of 40~53 m (excluding the depth near 47 m) is not as good as that of the targets located outside this range but not too poor to be accepted, while the resolution effect of the targets located near 47 m is too poor to be used. In addition, it can be found that the sign distribution of the reactive component is distance-dependent in the short range, so that it cannot be used in the target depth resolution.

#### 3.2.2. Relationship between Target Category-Resolution Accuracy and *SNR* in Single-Target Case

Target-radiated noise is assumed to be the superposition of the continuous spectrum and single-frequency line spectrum. The target moves away from the receiver after getting close to it. The detailed target simulation parameters are shown in Table 4 and Table 5. *SNR* is the abbreviation of Signal-Noise Ratio.

In the subsequent target category resolution, the parameter $P_{1}$ is defined as the proportion of the cases in which the sign is negative when discriminating the sign of the vertical complex acoustic intensity reactive component, $P_{1}=\frac{\sum_{i=1}^{N_{1}}L_{i}}{M_{1}N_{1}}\times 100\%$. $N_{1}$ is the total number of times of Monte Carlo simulation; $M_{1}$ is the total sampling numbers in each Monte Carlo simulation; $L_{i}$ ($i=1,2,\cdots ,N_{1}$) is the number of times of the cases in which the sign is negative when discriminating the sign of the vertical complex acoustic intensity reactive component in each simulation, $0\leq L_{i}\leq M_{1}$. The target category-resolution accuracy is expressed as $P_{2}=\{\begin{array}{cc} 1-P_{1} & \mathrm{surface}\;\mathrm{target}\\ P_{1} & \mathrm{underwater}\;\mathrm{target} \end{array}$.

**Figure 5:**
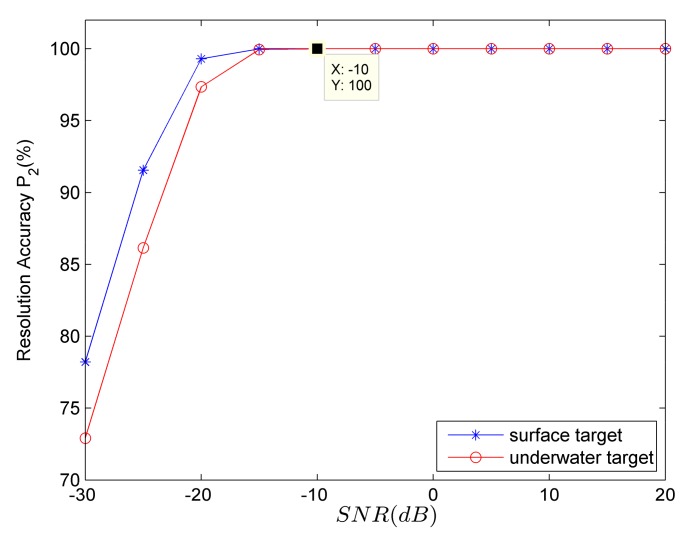
The relationship between target category-resolution accuracy and *SNR*.

Figure 5 shows the target category-resolution accuracies of surface (blue line marked with star symbol) and underwater (red line marked with circle symbol) targets using the 100th second data. It can be seen from Figure 5 that taking any processing period (100th second) within the whole time period, the accuracies $P_{2}$ are both greater than 70%. As *SNR* increases, $P_{2}$ gradually increases. When SNR≥−10 dB, $P_{2}$ all approach 100% which means the target category-resolution effect is rather good.

#### 3.2.3. Relationship between Target Azimuth-Estimation Accuracy and *SNR* in Single-Target Case

All the parameters are the same as shown in Table 4 and Table 5.

**Figure 6:**
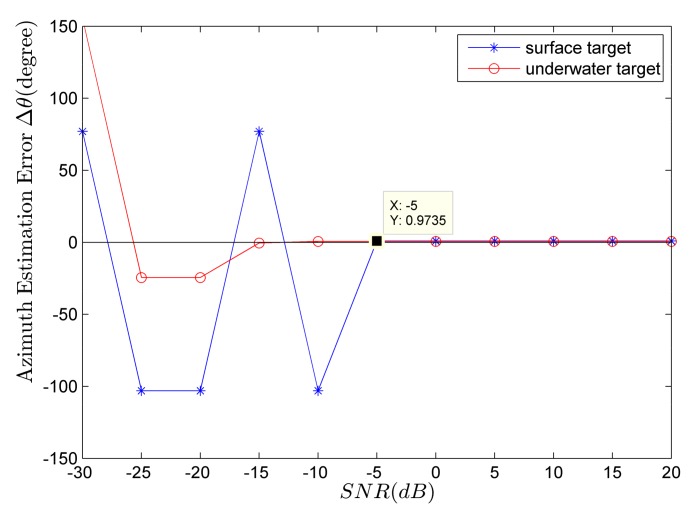
The relationship between target azimuth-estimation error and *SNR*.

Figure 6 shows the change of azimuth error with *SNR* in the single-target case. The blue line marked with a star symbol corresponds to the results of the single surface target and the red line marked with the circle symbol corresponds to the results of the single underwater target. From Figure 6, taking any time period (100th second), the azimuth-estimation errors of both surface and underwater targets are small enough for later calculations if SNR≥−5 dB.

### 3.3. Target Depth Resolution and Azimuth Estimation in Multi-Target Case

There are four targets in the simulation. Their platform velocity is $v_{s}=2\;m/s$. The target-radiated noise is assumed to be a combination of four single-frequency line spectrums whose frequencies are f=40 Hz, 48 Hz, 52 Hz, 56 Hz and the continuous spectrum. The first target moves away from the receiver after getting close to it. The other three targets move away from the receiver all the time. The target tonnages are all *DT* = 10,000 t, *SNR* = 0 dB. The sensor depth is 90 m, the total sailing time is 200 s. The other target simulation parameters are shown in Table 6, Table 7, Table 8 and Table 9.

**Figure 7:**
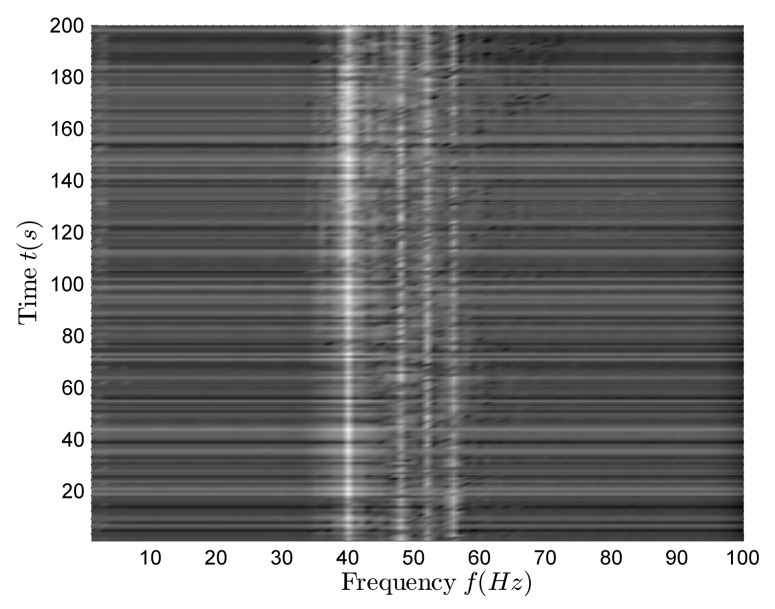
The time–frequency distribution of vertical complex acoustic intensity in the multi-target case.

Figure 7 shows the time–frequency distribution of the vertical complex acoustic intensity. There are four clear lines in Figure 7, and their corresponding frequencies are f=40,48,52,56 Hz.

**Figure 8:**
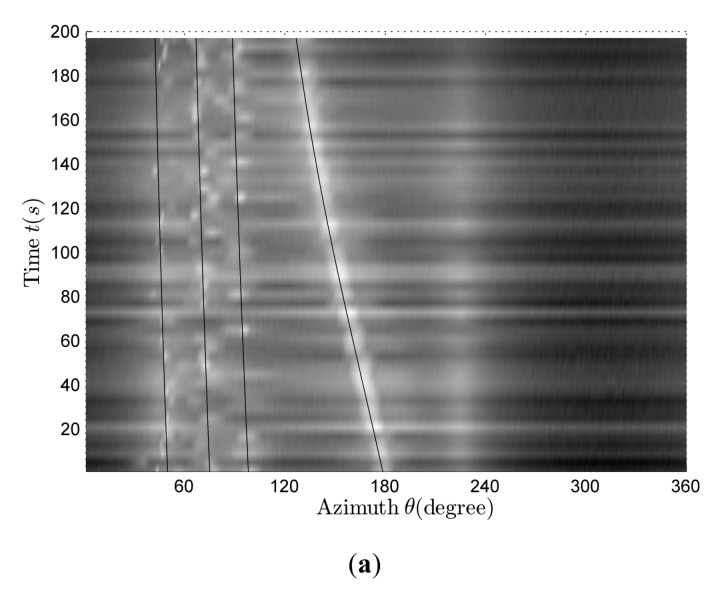

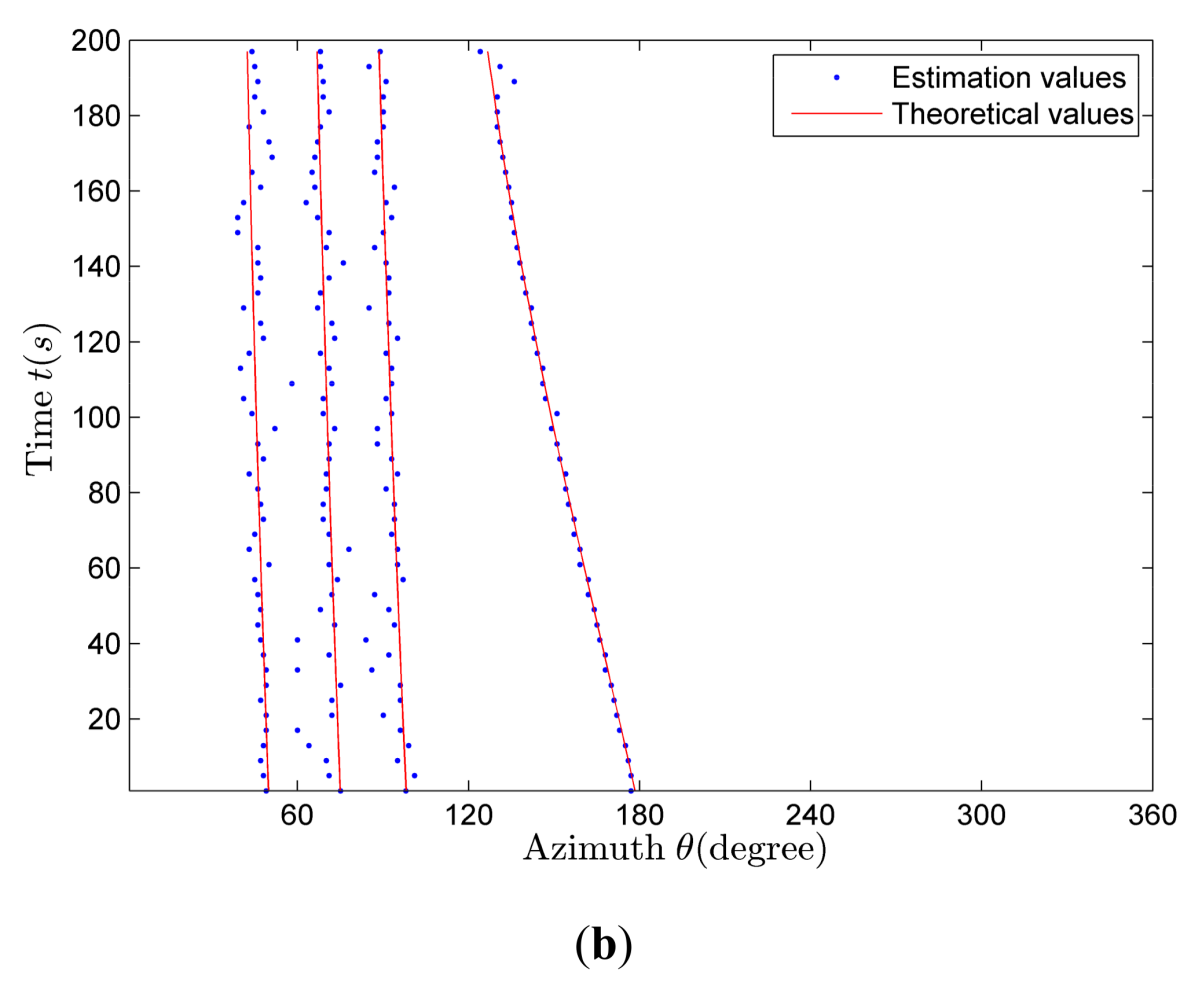
The bearing-time recording in the multi-target case. (**a**) Rough estimation; (**b**) accurate estimation.

Figure 8a is a rough estimation of the bearing-time recording at full frequency and the black line is the azimuth theoretical value reference curves corresponding to four line spectrums. There are four different curves for the azimuth variants in Figure 8a. By contrasting bearing-time recording information and azimuth theoretical curves, it can be found that only the fourth curve is close to its corresponding azimuth theoretical curve; there is a certain difference between the other three curves and their theoretical curves. With reference to Figure 7 and Figure 8, it can be seen that the received signal is composed of four different target-radiated signals. Using the algorithm described in Section 2.3.2 and Section 2.3.3, the accurate azimuth-estimation results shown in Figure 8b can be obtained. Comparing Figure 8a,b, we can find that the estimation results obtained by using the improved azimuth-estimation method proposed in this paper are more accurate. However, the original azimuth-estimation method has a rather good estimation effect only when the line spectrum intensity of the target signal is strong enough. As the line spectrum intensity becomes weaker, the corresponding azimuth-estimation effect becomes poorer.

Table 10 shows the target category accuracies in the multi-target case. As can be seen from Table 10, all the targets can be classified stably and correctly. The higher resolution accuracies of the first and second targets are mainly due to the fact that the line spectrum intensities of these two target signals are relatively higher. Although the target categories of the third and fourth targets are correctly identified, their resolution accuracies are relatively lower, mainly because the line spectrum intensities of these two target signals are relatively lower. These results confirm that there is a direct relationship between the target category-resolution accuracy and signal line spectrum intensity in the multi-target case. The stronger the line spectrum intensity, the higher the resolution accuracy.

### 3.4. Target Depth Resolution and Azimuth Estimation in the Single-Target Case

There is only one target in the simulation. The target radiated noise is assumed to be a combination of four single-frequency line spectrums and the continuous spectrum. The target moves away from the receiver after getting close to it. The other target simulation parameters are shown in Table 11.

**Figure 9:**
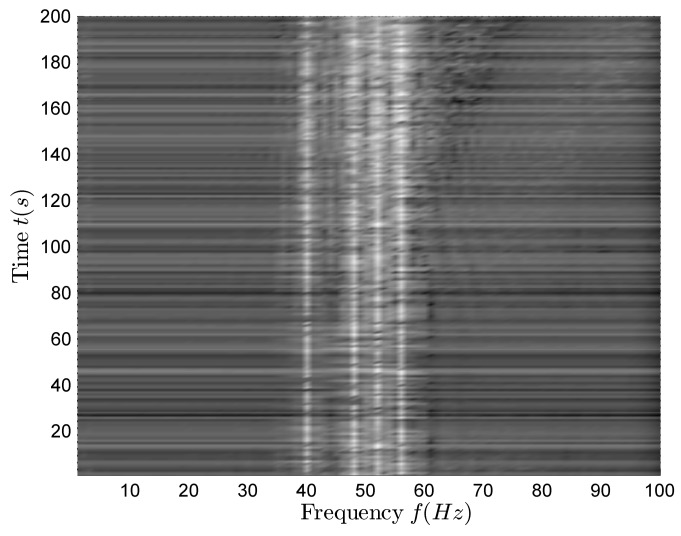
The time–frequency distribution of vertical complex acoustic intensity in the single-target case.

Figure 9 shows the time–frequency distribution of the vertical complex acoustic intensity. There are four clear lines in Figure 9, and their corresponding frequencies are f=40,48,52,56 Hz.

**Figure 10:**
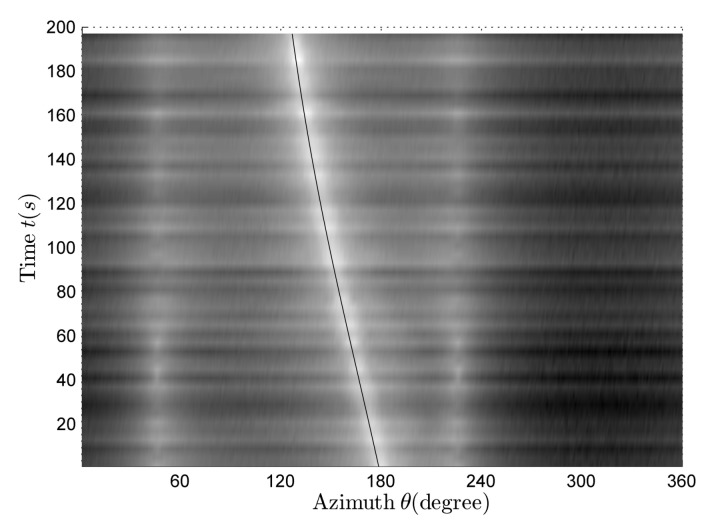
The rough estimation of bearing-time recording in the single-target case.

Figure 10 is a rough estimation of the bearing-time recording at full frequency and the black line is the azimuth theoretical curve. There is only one curve for the azimuth variants in Figure 10. With reference to Figure 9 and Figure 10, it can be seen that the received signal is radiated by one target.

Table 12 shows the target category-resolution accuracies in the single-target case. Using four different line spectrums of the same target signal for target category resolution, the target category-resolution accuracies are different, because the intensities of the four line spectrums are different. With the increase of the line spectrum intensity, the resolution effects become better. However, using any of the above four line spectrums, the targets can all be classified stably and accurately.

According to the results mentioned in Section 3.3 and Section 3.4, the proposed algorithm can successfully distinguish the multi-target multi-line spectrum case and the single-target multi-line spectrum case; moreover, the target number and category can be identified successfully. Furthermore, the improved azimuth-estimation method proposed in this paper is more suitable for azimuth estimation in the multi-target case, and the azimuth-estimation effect is better.

## 4. Sea Experiment Data and Results

The actual sea experiment was conducted to verify the practical feasibility of using the underwater sensor to identify the target category of the aerial targets. The specific experimental conditions are that a three-dimensional vector sensor is suspended underwater and connected to the underwater platform by the cable. Pressure and velocity signals radiated by an aerial target were collected by the vector sensor. The aerial target first approached the receiving sensor and then flew away from it at a constant velocity.

### 4.1. Azimuth- and Frequency-Estimation Results

**Figure 11:**
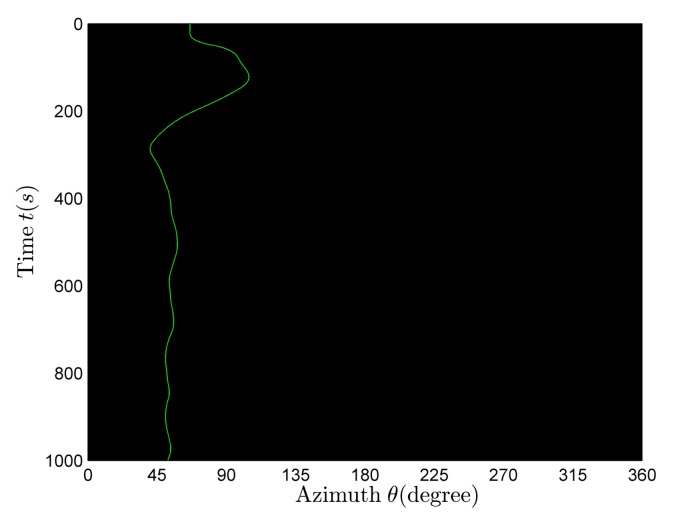
Azimuth-estimation results.

After the sea experiment data is processed, the azimuth and frequency estimation are carried out. The azimuth-estimation results are shown in Figure 11. The detailed estimation method is shown in [40].

**Figure 12:**
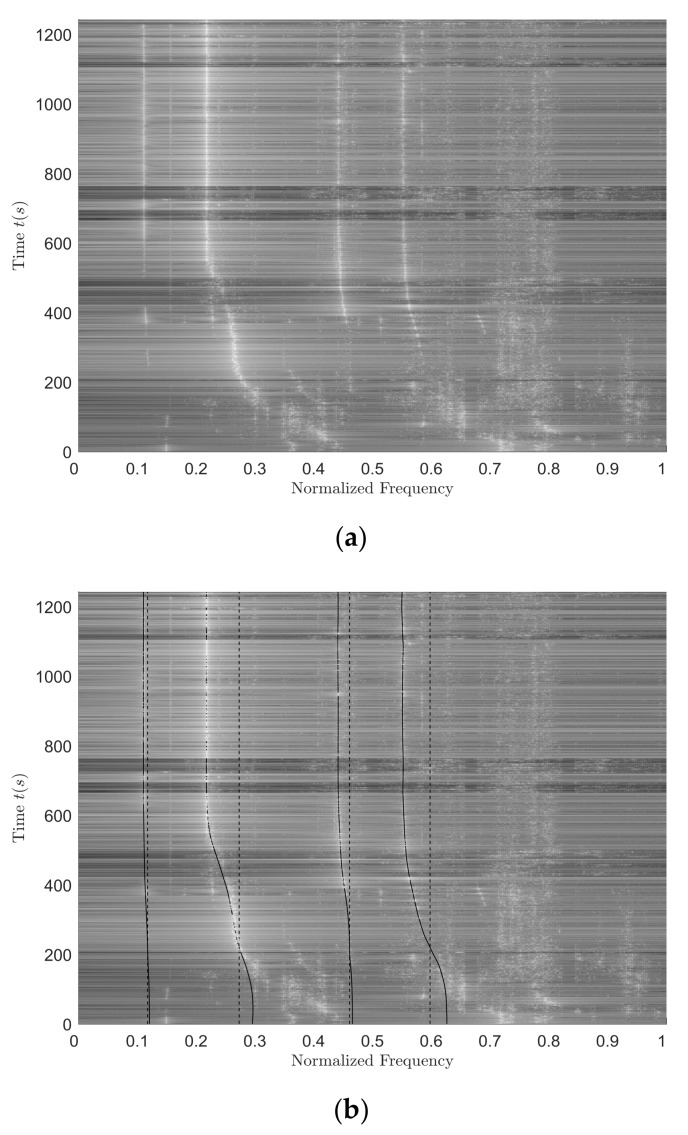
The normalized spectrum of the $v_{z}$ signal and its frequency sequence extraction results. (**a**) The normalized spectrum of the $v_{z}$ signal; (**b**) the frequency sequence extraction results.

Figure 12a shows the normalized spectrum of the $v_{z}$ signal. The reference value is the maximum frequency of the working band. The black solid lines in Figure 12b are the frequency sequence extraction results of the four clear-line spectrums in Figure 12a, and the black dotted lines are the source frequency reference lines corresponding to four line spectrums. Using the frequency sequence extraction results shown in Figure 12b, the source frequency estimation results can be obtained. Source frequency estimation values, true values and estimation errors are shown in Table 13. If the source frequency is estimated using data from the time period of the clearer line spectrum (namely, 800–1200 s), the corresponding estimation values, true values and estimation errors are shown in Table 14.

Comparing the corresponding values in Table 13 and Table 14, we can find that using the 800–1200 s data to estimate the source frequency, the source frequency estimation effects of the second, third and fourth line spectrums are relatively better. Although the estimation effect by using the first line spectrum is getting worse, the estimation error is still within the allowable range and the error value is rather small.

### 4.2. Target Category-Resolution Results

Target category resolution is performed using the sign distribution of the vertical complex acoustic intensity reactive component based on (15). The integral time lengths of spectrum estimation are 4, 6, 8, 10 s.

Based on the frequency extraction sequences corresponding to the four line spectrums in Figure 12b, using the data of 800–1200 s, target category resolution is performed. The resolution results are shown in Table 15. The percentage in the table indicates the proportion $P_{1}$ during the selected time period. In Section 2.2.2 and Section 3.1, the fact that an aerial target could be treated as the special case of a surface target during target category resolution has been verified through simulation. Therefore, the percentage in Table 15 is actually $P_{2}$. From the data in Table 15, it can be found that the target category-resolution effect using the frequency sequence of the first line spectrum is best. With the proper increase of the integral time length, the accuracy of the target resolution may improve. In the process of target resolution using the frequency extraction sequence of the second line spectrum, when the integration time length is short (4 s), the resolution results are not correct. With the increase of integral time length (6 s, 8 s, 10 s), the resolution effects are not ideal (resolution accuracies are low) although the resolution results are correct. Using the third and fourth line frequency extraction sequences, the target category cannot be accurately identified, mainly because these two line spectrums are not very clear in themselves, as shown in Figure 12a; namely, their line spectrum intensities are weak, resulting in resolution results that are not correct.

As can be seen from Table 15, it is feasible to classify aerial targets into surface targets as described in Section 2.2.2. Using the target height estimation method detailed in [40], if the receiving platform depth is known, we can get the distance between source and receiver; then the effective distinction between aerial targets and surface targets can be performed, so as to verify that with the use of the method proposed in this paper the aerial target depth resolution and the three-dimensional target depth resolution can be realized.

## 5. Conclusions

In this paper, we simulate the field of aerial and surface targets under the same environmental conditions. The theoretical derivation and simulation results can confirm that there is only amplitude difference between the aerial target field and the surface target field. An aerial target can be equivalent to a surface target in the target category resolution. Through the simulation, the relationship between the sign distribution of the vertical complex acoustic intensity reactive component and the depth is studied so that the target category resolution can be performed. The relationships between the accuracies of the target (surface targets and underwater targets) category resolution and *SNR* are studied by simulation. In this paper, an improved azimuth-estimation method is proposed, which is more suitable for multi-target cases and makes the azimuth-estimation results more ideal in multi-target cases. With the use of bearing-time recording information, the target number resolution can be realized. The relationship between the azimuth-estimation accuracy and *SNR* is studied. Comparing the depth-resolution and azimuth-estimation results of single-target multi-line spectrum and multi-target multi-line spectrum cases, it is proved that the proposed algorithm in this paper can distinguish between single-target multi-line spectrum and multi-target multi-line spectrum cases. Moreover, through processing of the actual sea experiment data, it is verified that the proposed algorithm is suitable for the aerial target depth resolution.

In this paper, we only verify the depth-resolution method of the aerial target and target number category-resolution method in the case of a single target through sea experiment data. At present, there is only a set of sea experiment data in the case of a single target. Firstly, the experimental data obtained by a three-dimensional vector sensor are difficult to acquire, so the proposed method is checked by only one experiment rather than several experiments. Then, the target number and category-resolution method in multi-target case proposed in this paper is currently only verified by the simulation and has not been verified by experimental data, because no funding project to support the acquisition of multi-target radiated noise collected by a single three-dimensional vector sensor is currently available. Finally, in the subsequent research process, we will try to continue to check the proposed method in this paper based on more actual experimental data processing so as to make the method more general and applicable in practice, and suggest an improved direction thereafter.

## Figures and Tables

**Table 1:** The marine environmental simulation parameters.

Parameters	Value	Parameters	Value
Sea depth *H*	100 m	Sound velocity in the air $c_{0}$	334 m/s
Water density $\rho_{1}$	$1.026\;{g/\mathrm{cm}}^{3}$	Sound velocity in the water $c_{1}$	1480 m/s
Seabed density $\rho_{2}$	$1.769\;{g/\mathrm{cm}}^{3}$	Sound velocity in the seabed $c_{2}$	1550 m/s

**Table 2:** Target simulation parameters.

Parameters	Value	Parameters	Value
Aerial target height	200 m	Equivalent surface target depth	5 m
Range of receiver depth	1~100 m	Range of horizontal distance	1~20 km

**Table 3:** Target simulation parameters.

Parameters	Value	Parameters	Value
Sensor depth	90 m	Target frequency	40 Hz
Range of target depth	1~100 m	Range of horizontal distance	1~20 km

**Table 4:** Target simulation parameters of surface target.

Parameters	Value	Parameters	Value
Target type	surface	Initial distance	1800 m
Target depth	5 m	Tonnage	10,000 t
Heading angle	$65^{\circ }$	Range of *SNR*	−30–20 dB
Target velocity	10 m/s	Target frequency	40 Hz
Platform velocity	2 m/s	Sensor depth	90 m
Closest distance	1650 m	Total sailing time	200 s

**Table 5:** Target simulation parameters of underwater target.

Parameters	Value	Parameters	Value
Target type	underwater	Initial distance	4100 m
Target depth	60 m	Tonnage	10,000 t
Heading angle	$30^{\circ }$	Range of *SNR*	−30–20 dB
Target velocity	8 m/s	Target frequency	52 Hz
Platform velocity	2 m/s	Sensor depth	90 m
Closest distance	2900 m	Total sailing time	200 s

**Table 6:** Target simulation parameters of the first target.

Parameters	Value	Parameters	Value
Target type	surface	Initial distance	1800 m
Target depth	5 m	Closest distance	1650 m
Heading angle	$65^{\circ }$	Target velocity	10 m/s

**Table 7:** Target simulation parameters of the second target.

Parameters	Value	Parameters	Value
Target type	surface	Initial distance	5000 m
Target depth	6 m	Closest distance	4000 m
Heading angle	$45^{\circ }$	Target velocity	9 m/s

**Table 8:** Target simulation parameters of the third target.

Parameters	Value	Parameters	Value
Target type	underwater	Initial distance	4100 m
Target depth	60 m	Closest distance	2900 m
Heading angle	$30^{\circ }$	Target velocity	8 m/s

**Table 9:** Target simulation parameters of the fourth target.

Parameters	Value	Parameters	Value
Target type	surface	Initial distance	3500 m
Target depth	8 m	Closest distance	2000 m
Heading angle	$15^{\circ }$	Target velocity	8 m/s

**Table 10:** Target category-resolution accuracy in multi-target case.

Target Serial Number	1	2	3	4
Accuracy $P_{2}$	99.8571%	95.1654%	91.0240%	89.7991%

**Table 11:** Target simulation parameters of surface target.

Parameters	Value	Parameters	Value
Target type	surface	Initial distance	1800 m
Target depth	5 m	Tonnage	10,000 t
Heading angle	$65^{\circ }$	*SNR*	0 dB
Target velocity	10 m/s	Target frequencies	40,48,52,56 Hz
Platform velocity	2 m/s	Sensor depth	90 m
Closest distance	1650 m	Total sailing time	200 s

**Table 12:** Target category-resolution accuracy in the single-target case.

Target Serial Number	1	2	3	4
Accuracy $P_{2}$	97.5492%	99.5842%	98.9678%	99.0210%

**Table 13:** The source frequency estimation results during the whole time period.

Serial Number of the Line Spectrum	1	2	3	4
Estimation value	0.1174	0.2728	0.4610	0.5975
True value	0.1167	0.2333	0.4667	0.5833
Estimation error (%)	0.60%	16.93%	1.22%	2.43%

**Table 14:** The source frequency estimation results obtained in the far field (800–1200 s).

Serial Number of the Line Spectrum	1	2	3	4
Estimation value	0.1103	0.2174	0.4414	0.5511
True value	0.1091	0.2182	0.4365	0.5456
Estimation error (%)	1.10%	0.37%	1.12%	1.01%

**Table 15:** Target category-resolution accuracy (800–1200 s).

	Serial Number of the Line Spectrum	1	2	3	4
Integral Time Length					
4 s		66.11%	46.39%	11.54%	17.79%
6 s		70.12%	54.70%	7.95%	21.45%
8 s		71.08%	53.98%	8.67%	23.13%
10 s		69.57%	53.62%	9.42%	27.78%
